# Outbreak of Severe Acute Respiratory Syndrome Coronavirus-2 B.1.620 Lineage in the General Hospital of Jeju Island, Republic of Korea

**DOI:** 10.3389/fmicb.2022.860535

**Published:** 2022-04-05

**Authors:** Young-Ran Ha, Een-suk Shin, Hyun-Jeong Kim, Eun-Hwa Hyeon, Jae-Sung Park, Yoon-Seok Chung

**Affiliations:** ^1^Jeju Branch Office, Honam Regional Center for Disease Control and Prevention, Korea Diseases Control and Prevention Agency, Jeju, South Korea; ^2^Division of Infectious Disease Diagnosis Control, Honam Regional Center for Disease Control and Prevention, Korea Diseases Control and Prevention Agency, Gwangju, South Korea; ^3^Jeju National Quarantine Station, Korea Diseases Control and Prevention Agency, Jeju, South Korea

**Keywords:** COVID-19, SARS-CoV-2, spike protein, Republic of Korea, lineage B.1.620

## Abstract

The number of coronavirus disease (COVID-19)-positive cases has increased in Jeju Island, Republic of Korea. Identification and monitoring of new mutations in severe acute respiratory syndrome coronavirus-2 (SARS-CoV-2) are extremely important to fighting the global pandemic. We report a breakout of the B.1.620 lineage, harboring the E484 mutation in the virus spike protein in a general hospital on Jeju Island. A cluster of cases was detected between August 4 and September 10, 2021, involving 20 patients positive for COVID-19 of 286 individuals exposed to the virus, comprising hospital patients, staff, and caregivers. We analyzed the epidemiological characteristics and spike proteins mutation sites using Sanger sequencing and phylogenetic analysis on these 20 patients. By analyzing genomic variance, it was confirmed that 12 of the confirmed patients harbored the SARS-CoV-2 B.1.620 lineage. The breakthrough rate of infection was 2% in fully vaccinated individuals among these patients. Next clade analysis revealed that these SARS-CoV-2 genomes belong to clade 20A. This is the first reported case of SARS-CoV-2 sub-lineage B.1.620, although the B.1.617.2 lineage has prevailed in August and September in Jeju, which has a geographical advantage of being an island. We reaffirm that monitoring the spread of SARS-CoV-2 variants with characteristic features is indispensable for controlling COVID-19 outbreaks.

## Introduction

Outbreaks of coronavirus disease (COVID-19) caused by severe acute respiratory syndrome coronavirus 2 (SARS-CoV-2) and its various lineages are being reported worldwide (Tegally et al., 2020; Hodcroft et al., 2021). The distinct SARS-CoV-2 lineages may affect transmissibility, diseases severity, and vaccine efficacy (Dudas et al., 2021). Here, we describe an outbreak of the B.1.620 lineage in Jeju Island, Republic of Korea.

The first case of B.1.620 was reported from Lithuania. It has also been reported from Central Africa and several European states, including France, Germany, and Spain (Dudas et al., 2021; Singh et al., 2021). In Republic of Korea, B.1.620 was imported from Kenya and Malawi in March, 2021 (Park et al., 2022). B.1.620 carries 26 mutations and numerous deletions (Dudas et al., 2021) in its genomes, which are different from that of the reference Wuhan Hu-1 strain (Rambaut et al., 2020; O’Toole et al., 2021). In the Pangolin nomenclature, these genomes are assigned to clade 20A (Rambaut et al., 2020; Pirnay et al., 2021). They also carry the D614G mutation, which promotes the infectivity of SARS-CoV-2 by enhancing its interactions with the host angiotensin-converting enzyme 2 (ACE2) receptor *via* increasing the affinity of its receptor-binding domain (RBD) for ACE2 (Hou et al., 2020; Korber et al., 2020; Yurkovetskiy et al., 2020; Plante et al., 2021). The E484K mutation in the RDB occurs at the periphery of the RDB–ACE2 interface and may introduce new salt bridges with E35/E75 of ACE2 (Lan et al., 2020; Dudas et al., 2021). Therefore, B.1.620 may escape antibody-mediated immunity *via* interaction with ACE2 (Dudas et al., 2021). A previous study reported that B.1.620 is found 2.4 times in vaccine breakthrough cases compared with its population prevalence (Dudas et al., 2021; Šimaitis, 2021).

B.1.620 was formerly variants under monitoring (VUM) as of July 14, 2021 but was reclassified as formerly monitored variants on November 9, 2021 (WHO, 2021). Formerly monitored variants must meet at least one of the following criteria: (i) no longer spreading at the level of global public health significance, (ii) have been circulating over the long term without any epidemiological concern, or (iii) do not have any concerning properties based on scientific evidence (WHO, 2021).

In this study, we describe the outbreak of the SARS-CoV-2 B.1.620 lineage, the first outbreak in the general hospital of Jeju Island, Republic of Korea. What remains unknown is the influence of B.1.620 lineage on transmission dynamics in 286 individuals, comprising staff, patients, and caregivers, who may or may not be fully vaccinated. Therefore, we describe the epidemiological profile, including vaccination status, type, and symptoms. Over 10% of polymerase chain reaction (PCR)-positive SARS-CoV-2 cases were sequenced and analyzed phylogeny of B.1.620 lineage compared with other SARS-CoV-2 B.1.620 cases in Jeju Island.

## Materials and Methods

### RNA Isolation and Reverse Transcription (RT)-PCR

COVID-19-positive specimens were provided by the Jeju Special Self-Governing Province Institute of Environment Research. The specimens were handled in a Class II biosafety cabinet (Thermo scientific 1300 series A2) at a biosafety level 2 (BL2) laboratory. RNA was isolated using an RNA extraction kit (Qiagen, Valencia, CA, United States) according to the manufacturer’s instructions. cDNA was synthesized using SuperScript IV First-Strand Synthesis System (Invitrogen, Carlsbad, CA, United States). PCR was conducted using PrimeSTAR GXL DNA Polymerase (Takara, Shiga, Japan) from February to August, 2021. RT-PCR was conducted using a DiaStar 2 × OneStep RT-PCR premix kit (SolGent, Daejeon, South Korea) and a SEQMAX qPCR one-step master mix (Nine Korea, South Korea). Sequences of the primers used for RT-PCR are shown in Table 1. To detect mutations of SARS-CoV-2 spike protein, L71/R75, L76/R79, L80/R84 primer sets were used to conduct RT-PCR, independently. RT-PCR was performed under the following conditions: an initial reverse transcription step at 50°C for 30 min followed by a denaturation step at 95°C for 5 to 15 min. This was followed by 35 cycles of 30 s at 95°C, 30 s at 58°C, 1 min 30 s at 68°C, and a final extension step at 68°C for 7 min.

**TABLE 1:** A list of primer sequences used for RT-PCR analysis and sanger sequencing of spike protein.

Fragment	Primer	Primer sequence	Binding position	Fragment size (bp)
**Fragment 1**	L71	Forward: 5’-ACAAATCCAATTCAGTTGTCTTCCTATTC-3′	21,358-21,386	1,546
	L73	Forward: 5′-CAATTTTGTAATGATCCATTTTTGGGTGT-3′	21,962-21,990	
	R73	Reverse: 5′-CACCAGCTGTCCAACCTGAAGA-3′	22,325-22,346	
	R75	Reverse: 5′-ACCACCAACCTTAGAATCAAGATTGT-3′	22,878-22,903	
**Fragment 2**	L76	Forward: 5′-AGGGCAAACTGGAAAGATTGCT-3′	22,798-22,819	1,372
	L78	Forward: 5′-CAACTTACTCCTACTTGGCGTGT-3′	23,444-23,466	
	R77	Reverse: 5′-CAGCCCCTATTAAACAGCCTGC-3′	23,501-23,522	
	R79	Reverse: 5′-CATTTCATCTGTGAGCAAAGGTGG-3′	24,146-24,169	
**Fragment 3**	L80	Forward: 5′-TTGCCTTGGTGATATTGCTGCT-3′	42,079-24,100	1,595
	L83	Forward: 5′-TCCTTTGCAACCTGAATTAGACTCA-3′	24,979-25,003	
	R82	Reverse: 5′-TGCCAGAGATGTCACCTAAATCAA-3′	25,053-25,076	
	R84	Reverse: 5′-AGGTGTGAGTAAACTGTTACAAACAAC-3′	25,647-25,673	

*The position number of each primer sequence was compared to Wuhan Wuhan Hu-1, genome sequence (accession number: NC_045512.2).*

### DNA Sequencing and Analysis

DNA sequencing was performed using a standard protocol (Cosmo Genetech, Seoul, Korea). To analyze the sequences, L71/R75, L76/R79, L80/R84 and inner primers, including L73/R73, L78/R77, L83/R82, were used (Table 1). With sequence data from the COVID-19-positive specimens in Jeju Island, full-length sequences of SARS-CoV-2 spike proteins from different geographical origins were downloaded in FASTA format from GISAID^1^. The non-coding 3′ and 5′ regions were trimmed using CLC Genomic Workbench 5.0.1 software (CLC bio, Denmark). Multiple sequence alignment was performed using the MAFFT algorithm. Phylogenetic analysis was performed using the maximum likelihood method using Molecular Evolutionary Genetics Analysis version 10.2.5 (MEGA X). Branch support was calculated using bootstrapping, consisting of 1,000 alignments. SARS-CoV-2 B.1.620 lineage genomes from the outbreak cases of the hospital in Jeju Island were classified using Nextclade^2^. The hCoV-19 genome sequences of B.1.620 strain in Jeju were deposited in GISAID (Table 2).

**TABLE 2:** Spike protein mutations with their locations identified using 12 sequences of general hospital cases.

Accession ID in GISAID	Length (nt)	ExistingMutList	Deletions
EPI_ISL_9167163	3804	(Spike_H245Y,Spike_P681H,Spike_D614G,Spike_Y144del,Spike_ L244del,Spike_V126A,Spike_S477N,Spike_E484K,Spike_T1027I, Spike_D1118H,Spike_L242del,Spike_A243del,Spike_V70del,Spike_H69del)	21765-21770,21992-21994,22283-22291
EPI_ISL_9167164	3807	(Spike_P26S,Spike_H245Y,Spike_P681H,Spike_D614G,Spike_ L244del,Spike_V126A,Spike_S477N,Spike_E484K,Spike_T1027I, Spike_D1118H,Spike_L242del,Spike_A243del,Spike_V70del,Spike_H69del)	21765-21770,21992-21994,22283-22291
EPI_ISL_9167165	3807	(Spike_P26S,Spike_H245Y,Spike_P681H,Spike_D614G,Spike_ L244del,Spike_V126A,Spike_S477N,Spike_E484K,Spike_T1027I, Spike_D1118H,Spike_L242del,Spike_A243del,Spike_V70del,Spike_H69del)	21765-21770,21992-21994,22283-22291
EPI_ISL_9167166	3804	(Spike_P26S,Spike_H245Y,Spike_P681H,Spike_D614G,Spike_Y144del,Spike_ L244del,Spike_V126A,Spike_S477N,Spike_E484K,Spike_T1027I, Spike_D1118H,Spike_L242del,Spike_A243del,Spike_V70del,Spike_H69del)	21765-21770,21992-21994,22283-22291
EPI_ISL_9173037	3806	(Spike_P26S,Spike_H245Y,Spike_P681H,Spike_D614G,Spike_Y144del,Spike_ L244del,Spike_V126A,Spike_S477N,Spike_E484K,Spike_T1027I, Spike_D1118H,Spike_L242del,Spike_A243del,Spike_V70del,Spike_H69del)	21765-21770,22283-22291
EPI_ISL_9167167	3804	(Spike_P26S,Spike_H245Y,Spike_P681H,Spike_D614G,Spike_Y144del,Spike_ L244del,Spike_V126A,Spike_S477N,Spike_E484K,Spike_T1027I, Spike_D1118H,Spike_L242del,Spike_A243del,Spike_V70del,Spike_H69del)	21765-21770,21992-21994,22283-22291
EPI_ISL_9167168	3804	(Spike_P26S,Spike_H245Y,Spike_P681H,Spike_D614G,Spike_Y144del,Spike_ L244del,Spike_V126A,Spike_S477N,Spike_E484K,Spike_T1027I, Spike_D1118H,Spike_L242del,Spike_A243del,Spike_V70del,Spike_H69del)	21765-21770,21992-21994,22283-22291
EPI_ISL_9167169	2575	(Spike_P26S,Spike_H245Y,Spike_P681H,Spike_D614G,Spike_Y144del,Spike_ L244del,Spike_V126A,Spike_S477N,Spike_E484K,Spike_L242del,Spike_ A243del,Spike_V70del,Spike_H69del)	21765-21770,21995-21996,22283-22291
EPI_ISL_9167170	2577	(Spike_P26S,Spike_H245Y,Spike_P681H,Spike_D614G,Spike_Y144del,Spike_ L244del,Spike_V126A,Spike_S477N,Spike_E484K,Spike_L242del,Spike_ A243del,Spike_V70del,Spike_H69del)	21765-21770,22283-22291
EPI_ISL_9167171	2576	(Spike_P26S,Spike_H245Y,Spike_P681H,Spike_D614G,Spike_Y144del,Spike_ L244del,Spike_V126A,Spike_S477N,Spike_E484K,Spike_L242del,Spike_ A243del,Spike_V70del,Spike_H69del)	21765-21770,21992-21994,22283-22291
EPI_ISL_9173038	1966	(Spike_P26S,Spike_H245Y,Spike_N148Q,Spike_D614G,Spike_Y145P,Spike_ L244del,Spike_H146Q,Spike_S477N,Spike_N149Q,Spike_E484K,Spike_ L242del,Spike_A243del,Spike_V70del,Spike_H69del)	21765-21770,21992-21994,22283-22291
EPI_ISL_9167172	1773	(Spike_P26S,Spike_H245Y,Spike_D614G,Spike_Y144del,Spike_ L244del,Spike_V126A,Spike_S477N,Spike_E484K,Spike_L242del,Spike_ A243del,Spike_V70del,Spike_H69del)	21765-21770,21992-21994,22283-22291

### Calculation of Infection Attack Rate (IAR)

We calculated the attack rate in 286 individuals of a hospital by vaccination status. We estimated 95% confidence interval (CI) and relative risk (RR), which is the ratio of the attack rates of a disease among vaccinated and not-vaccinated individuals.

## Results

### Epidemiological Characteristics of the COVID-19 Outbreak Cases

A cluster of cases occurred in the general hospital in Jeju Island between August 4 and September 10, 2021. This cluster included 286 individuals, comprising staff, patients, and caregivers of the hospital. Among them, 20 people, that is, 8 patients, 8 caregivers, and 4 family caregivers, were confirmed to be COVID positive by September 2 (Table 3). Among general hospital individuals exposed to the virus, 12.9% (37) had received their first dose of COVID-19 vaccine, 69.9% (200) were fully vaccinated, and 17.1% (49) were not vaccinated. The vaccinated individuals had received the AstraZeneca, Pfizer-BioNTech, Moderna, or Janssen vaccine. Among the fully vaccinated persons, 60.8% (174) were AstraZeneca vaccine recipients, 7.3% (21) had received the Pfizer-BioNTech vaccine, and 1.7% (5) were Moderna vaccine recipients and were vaccinated 14 days before the outbreak. Therefore, the breakthrough rate of infection was 2% in completing primary COVID-19 vaccination. Of the 20 positive patients, 7 had been vaccinated one time, 6 were completely vaccinated, and 7 had not been vaccinated. The most common symptoms of the positive patients were shiver/chills (20%), cough (10%), sore throat (5%), muscle ache (5%). Thirteen patients did not show any symptoms (Table 3).

**TABLE 3:** Epidemiological characteristics of confirmed cases for the COVID-19 outbreak at the general hospital in Jeju Island, Republic of Korea, in August 2021.

Characteristics		COVID-19 case (*n* = 20)		Non-COVID-19 case (*n* = 266)		Total (*n* = 286)	
				
		*N*	%	*N*	%	*n*	%
**Case**		20	7.0%	266	93.0%	286	100.0%
Staff	Healthcare workers	0	0.0%	68	23.8%	68	23.8%
	etc. (caregivers)	8	2.8%	138	48.3%	146	51.0%
Participant	Patients	8	2.8%	42	14.7%	50	17.5%
	(unpaid) Family caregivers	4	1.4%	18	6.3%	22	7.7%
**Sex**							
Male		8	2.8%	109	38.1%	117	40.9%
Female		12	4.2%	157	54.9%	169	59.1%
**Age group (years)**							
≤ 30		2	0.7%	101	35.3%	103	36.0%
40-49		0	0.0%	49	17.1%	49	17.1%
50-59		4	1.4%	49	17.1%	53	18.5%
60-69		6	2.1%	38	13.3%	44	15.4%
70-79		7	2.4%	18	6.3%	25	8.7%
≥ 80		1	0.3%	11	3.8%	12	4.2%
**Vaccination status**							
Unvaccination		7	2.4%	42	14.7%	49	17.1%
Incompleted vaccination		8	2.8%	29	10.1%	37	12.9%
Completed vaccination		5	1.7%	195	68.2%	200	69.9%
**Symptoms**							
Asymptomatic		13	65.0%	-	-	-	-
Symptomatic	Cough	2	10.0%	-	-	-	-
	Sore throat	1	5.0%	-	-	-	-
	Muscle ache	1	5.0%	-	-	-	-
	Nasal obstruction	1	5.0%	-	-	-	-
	Shivers/Chills	4	20.0%	-	-	-	-

The attack rate was 14.3 and 5.5% in the not-vaccinated and vaccinated individuals, respectively. The relative risk (RR) was 5.5% in vaccinated individuals. The vaccinated individuals were lower than unvaccinated individuals (RR < 1, 95% confidence interval < 0.02). In addition, the total vaccine effectiveness against infection was 96.2%. The vaccine effectiveness was 84.9 and 98.3% in the first dose of COVID-19 vaccine and fully vaccinated individuals, respectively.

### Phylogenetic Analysis of SARS-CoV-2 B.1.620 Lineage From the Outbreak Cases

**FIGURE 1:**
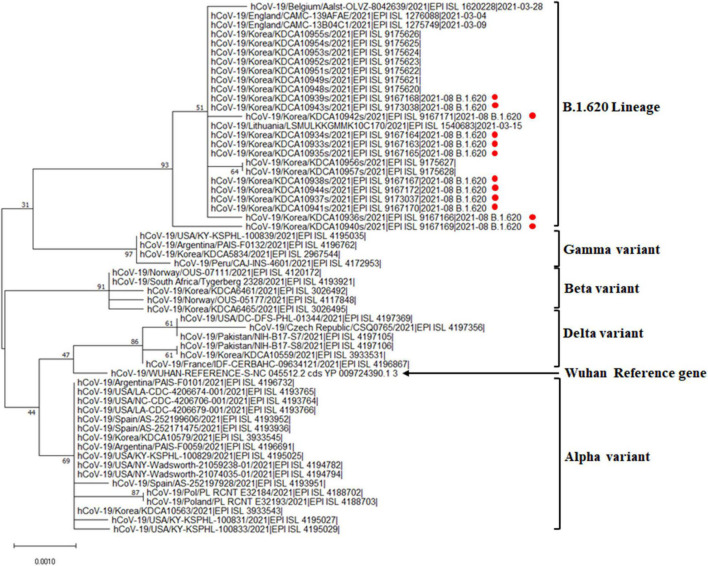
Phylogenetic analysis of the SARS-CoV-2 B.1.620 lineage in the general hospital of Jeju Island. Phylogenetic analysis of alpha, beta, gamma, delta variants, and B.1.620 lineage with outbreak cases in Jeju Island. Red circles indicate the genomes from hospital outbreak cases in Jeju Island.

The genome sequences obtained were compared with the reference SARS-CoV-2 isolate, Wuhan Hu-1, genome sequence (accession number: NC_045512.2). The hospital outbreak strains clustered closely around the other B.1.620 lineages in Jeju Island (Figure 1). The group of B.1.620 sequences was closer to the root of the B.1.1.7 lineage (i.e., the alpha variant) than to B.1.351 (the beta variant), P.1 (the gamma variant), and B.1.617.2 (the delta variant). The phylogenetic tree of B.1.620 lineages was composed of the outbreak cases (EPI_ISL_9167163 ∼ EPI_ISL_9167172, EPI_ISL_9173037, and EPI_ISL_9173038) and the other cluster in Jeju Island (EPI_ISL_9175620 ∼ EPI_ISL_9175628). SARS-CoV-2 sequences belonging to the B.1.620 lineages, which were detected in Jeju, were closely related to each other. In addition, we included the B.1.620 lineage, such as hCoV-19/Lithuania/LSMULKKGMMK10C170/2021 (EPI_ISL_1540683), hCoV-19/England/CAMC-13B04C1/2021 (EPI_ISL_1275749), Belgium/Aalst-OLVZ-8042639/2021 (EPI_ISL_1620228), and England/CAMC-139AFAE/2021 (EPI_ISL_1276088). The Nextclade analysis revealed that SARS-CoV-2 genomes from outbreak cases of hospitals in Jeju belong to clade 20A (Supplementary Table 1). Among clade 20A, B.1.620 lineages of Jeju closed with the other B.1.620 lineages, such as hCoV-19/Lithuania/LSMULKKGMMK10C170/2021 (EPI_ISL_1540683), hCoV-19/England/CAMC-13B04C1/2021 (EPI_ISL_1275749), and England/CAMC-139AFAE/2021 (EPI_ISL_1276088) (Figure 1).

## Conclusion

We described the first outbreak of SARS-CoV-2 lineage B.1.620 in Jeju Island. B.1.620 has been associated with many VOC-like mutations and deletions. Our phylogenetic analysis revealed that the cluster of hospital outbreaks was closed compared with those of other variants. Furthermore, the clustering pattern with branching suggested that this B.1.620 lineage hospital outbreak could indicate community spread with the other B.1.620 lineages in Jeju from April to September 2021.

B.1.620 was introduced to Jeju on April 27, 2021. After introduction, the proportion of B.1.620 increased from April (2.1%) to June (18.9%) (Supplementary Figure 1). However, B.1.617.2 (the delta variant) has become dominant since July (59.3%) to September (96.8%) in Jeju Island (Supplementary Figure 2). Interestingly, the hospital outbreak of the B.1.620 lineage occurred in August and September 2021, although the B.1.617.2 lineage prevailed all over Jeju Island.

Hospitals could be the susceptible place to outbreaks because of highly transmissible variants with less-effective vaccines (Susky et al., 2021). In this study, the outbreak in the hospital of Jeju might contain several epidemiological aspects of learnings. First, most of COVID-19 sub-lineage B.1.620 positive cases were asymptomatic or have only mild symptoms. Secondly, despite vaccination, hospital exposers from the virus may result in transmission of the B.1.620 lineage. Thus, we conclude that continuous monitoring of COVID-19 cases and SARS-CoV-2 variants in Jeju Island is essential for controlling virus transmission and subsequent outbreaks.

## Data Availability Statement

The datasets presented in this study can be found in online repositories. The names of the repository/repositories and accession number(s) can be found below: https://www.gisaid.org/, EPI_ISL_9167163 – EPI_ISL_9167172; EPI_ISL_9173037; and EPI_ISL_9173038.

## Author Contributions

Y-RH contributed to the conception, design, data acquisition, and drafting of the manuscript. E-SS participated in epidemiological surveys. H-JK and E-HH conducted the experiment. J-SP advised the experimental procedure and treatment of specimens. Y-SC conceived the entire study and helped draft the manuscript. All authors read and approved the final manuscript.

## Conflict of Interest

The authors declare that the research was conducted in the absence of any commercial or financial relationships that could be construed as a potential conflict of interest.

## Publisher’s Note

All claims expressed in this article are solely those of the authors and do not necessarily represent those of their affiliated organizations, or those of the publisher, the editors and the reviewers. Any product that may be evaluated in this article, or claim that may be made by its manufacturer, is not guaranteed or endorsed by the publisher.

## Footnotes

1
https://www.gisaid.org/

2
http://clades.nextstrain.org/

## References

[B1] Dudas G. Hong S. L. Potter B. I. Calvignac-Spencer S. Niatou-Singa F. S. Tombolomako T. B. (2021). Emergence and spread of SARS-CoV-2 lineage B.1.620 with variant of concern-like mutations and deletions. *Nat. Commun.* 12:5769. 10.1038/s41467-021-26055-8 34599175 PMC8486757

[B2] Hodcroft E. B. Zuber M. Nadeau S. Vaughan T. G. Crawford K. H. D. Althaus C. L. (2021). Spread of a SARS-CoV-2 variant through Europe in the summer of 2020. *Nature* 595 707–712. 34098568 10.1038/s41586-021-03677-y

[B3] Hou Y. J. Chiba S. Halfmann P. Ehre C. Kuroda M. Dinnon K. H. (2020). SARS-CoV-2 D614G variant exhibits efficient replication ex vivo and transmission in vivo. *Science* 370 1464–1468. 10.1126/science.abe8499 33184236 PMC7775736

[B4] Korber B. Fischer W. M. Gnanakaran S. Yoon H. Theiler J. Abfalterer W. (2020). Tracking changes in SARS-CoV-2 spike: evidence that D614G increases infectivity of the COVID-19 virus. *Cell* 182:e819. 10.1016/j.cell.2020.06.043 32697968 PMC7332439

[B5] Lan J. Ge J. Yu J. Shan S. Zhou H. Fan S. (2020). Structure of the SARS-CoV-2 spike receptor-binding domain bound to the ACE2 receptor. *Nature* 581 215–220. 10.1038/s41586-020-2180-5 32225176

[B6] O’Toole A. Scher E. Underwood A. Jackson B. Hill V. Mccrone J. T. (2021). Assignment of epidemiological lineages in an emerging pandemic using the pangolin tool. *Virus Evol.* 7:veab064. 10.1093/ve/veab064 34527285 PMC8344591

[B7] Park A. K. Kim I. H. Man Kim H. Lee H. Lee N. J. Kim J. A. (2022). SARS-CoV-2 B.1.619 and B.1.620 lineages. South Korea, 2021. *Emerg. Infect. Dis.* 28 415–419. 10.3201/eid2802.211653 35076365 PMC8798691

[B8] Pirnay J. P. Selhorst P. Hong S. L. Cochez C. Potter B. Maes P. (2021). Variant analysis of SARS-CoV-2 genomes from belgian military personnel engaged in overseas missions and operations. *Viruses* 13:1359. 10.3390/v13071359 34372565 PMC8310367

[B9] Plante J. A. Liu Y. Liu J. Xia H. Johnson B. A. Lokugamage K. G. (2021). Author correction: spike mutation D614G alters SARS-CoV-2 fitness. *Nature* 595:E1. 10.1038/s41586-021-03657-2 34131306 PMC8205212

[B10] Rambaut A. Holmes E. C. O’toole A. Hill V. Mccrone J. T. Ruis C. (2020). A dynamic nomenclature proposal for SARS-CoV-2 lineages to assist genomic epidemiology. *Nat. Microbiol.* 5 1403–1407. 10.1038/s41564-020-0770-5 32669681 PMC7610519

[B11] Šimaitis A. (2021). *A Situation Report to the Lithuanian Government Regarding SARS-CoV-2 [Online].* Available online at: https://lrv.lt/uploads/main/documents/files/20210511%20COVID-19%20situacijos%20ap%C5%BEvalga.pdf (accessed November 18, 2021).

[B12] Singh D. D. Parveen A. Yadav D. K. (2021). SARS-CoV-2: emergence of new variants and effectiveness of vaccines. *Front. Cell Infect. Microbiol.* 11:777212. 10.3389/fcimb.2021.777212 34970509 PMC8713083

[B13] Susky E. K. Hota S. Armstrong I. E. Mazzulli T. Kestenberg S. Casaubon L. K. (2021). Hospital outbreak of the severe acute respiratory coronavirus virus 2 (SARS-CoV-2) delta variant in partially and fully vaccinated patients and healthcare workers in Toronto, Canada. *Infect. Control Hosp. Epidemiol.* 28 1–4. 10.1017/ice.2021.471 34706787 PMC8593380

[B14] Tegally H. Wilkinson E. Giovanetti M. Iranzadeh A. Fonseca V. Giandhari J. (2020). Emergence and rapid spread of a new severe acute respiratory syndrome-related coronavirus 2 (SARS-CoV-2) lineage with multiple spike mutations in South Africa. *medRxiv* [Preprint] 10.1101/2020

[B15] WHO (2021). *Tracking SARS-CoV-2 Variants [Online].* Available online at: https://www.who.int/en/activities/tracking-SARS-CoV-2-variants/ (accessed November 10, 2021).

[B16] Yurkovetskiy L. Wang X. Pascal K. E. Tomkins-Tinch C. Nyalile T. P. Wang Y. (2020). Structural and functional analysis of the D614G SARS-CoV-2 spike protein variant. *Cell* 183:e738. 10.1016/j.cell.2020.09.032 PMC7492024 32991842

