# Supplementary material for: Outbreak of Severe Acute Respiratory Syndrome Coronavirus-2 B.1.620 Lineage in the General Hospital of Jeju Island, Republic of Korea
Source: Front Microbiol. 2022 Apr 5;13:860535. doi: 10.3389/fmicb.2022.860535 (PMC9037150; doi:10.3389/fmicb.2022.860535)
Supplement: Supplementary file 1 [file Data_Sheet_1.docx]

Supplementary Material

***Sanger sequencing and analysis***

COVID-19-positive specimens were provided by the Jeju Special Self-Governing Province Institute of Environment Research. PCR was conducted using PrimeSTAR GXL DNA Polymerase (Takara, Shiga, Japan). RT-PCR was conducted using a DiaStar 2× OneStep RT-PCR premix kit (SolGent, Daejeon, South Korea) and SEQMAX qPCR one-step master mix (Nine Korea, South Korea). To detect mutations of SARS-CoV-2 spike protein, L71/R75, L76/R79, L80/R84 primer sets were used to conduct RT-PCR, independently (Table 3). RT-PCR was performed under the following conditions: an initial reverse transcription step at 50 °C for 30 min followed by a denaturation step at 95 °C for 5 to 15 min. This was followed by 35 cycles of 30 s at 95 °C, 30 s at 58 °C, 1 min 30 s at 68 °C, and a final extension step at 68 °C for 7 min. DNA sequencing was performed using a standard protocol (Cosmo Genetech, Seoul, Korea). To analyze the sequences, L71/R75, L76/R79, L80/R84 and inner primers including L73/R73, L78/R77, L83/R82 were used (Table 3). The non-coding 3' and 5' regions were trimmed and analyzed using CLC Genomic Workbench 5.0.1 software (CLC bio, Denmark). In this study, we analyzed the number of variants from February to September (Supplementary Figure 1 and 2).

Clade 20A with SARS-CoV-2 B.1.620 lineage genomes from the outbreak cases of the hospital on Jeju Island were classified using Nextclade (<http://clades.nextstrain.org/>) (Supplementary Figure 3). In this study, included the information of mutations and data were analyzed using Nextclade as shown in supplementary Table 1.


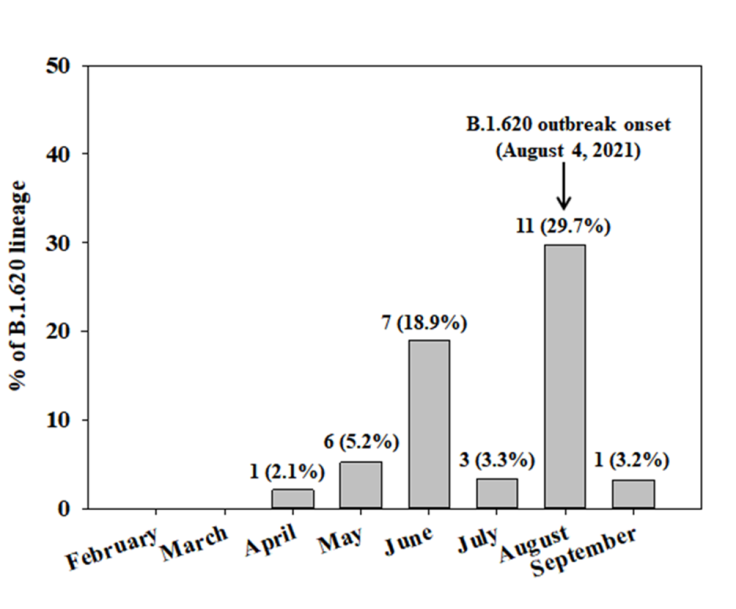


**Supplementary Figure 1.** Proportion of the B.1.620 lineage from February to September 2021 on Jeju Island among analyzed cases.


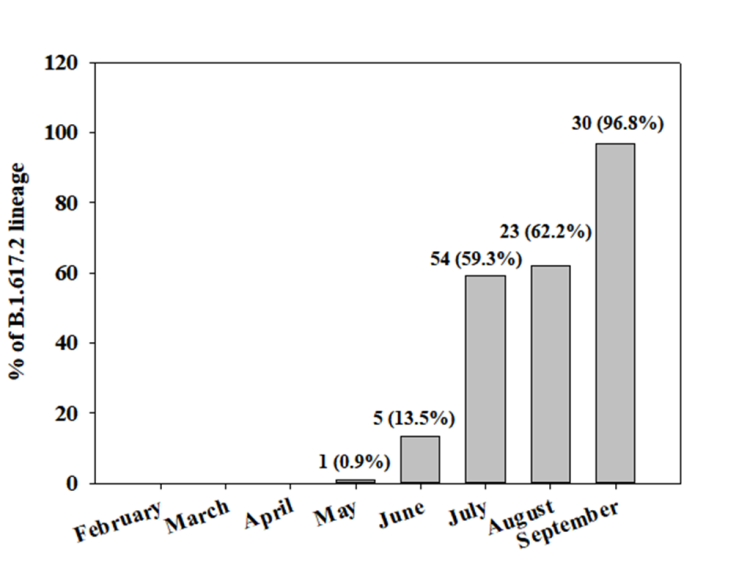


**Supplementary Figure 2.** Proportion of B.1.617.2 lineage from February to September 2021 on Jeju Island among analyzed cases


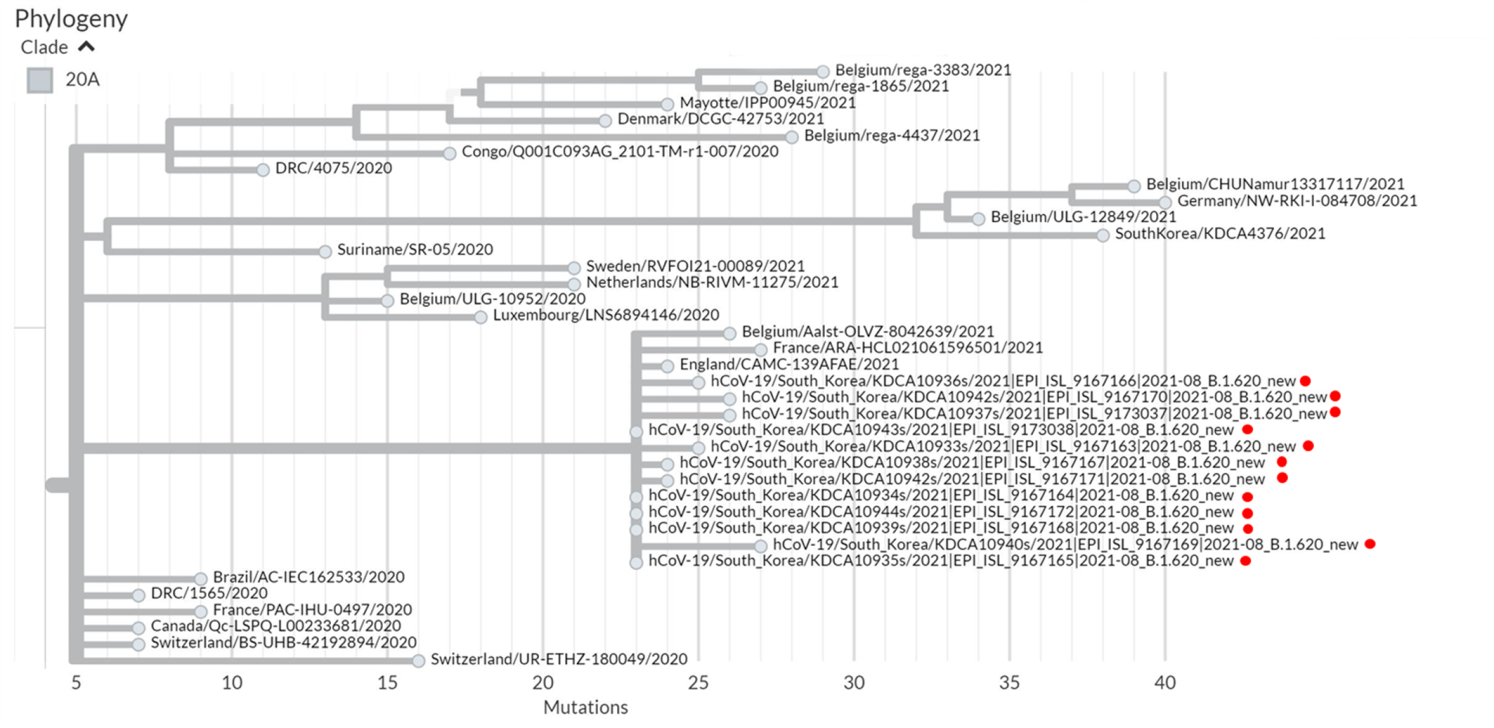


**Supplementary Figure 3.** Phylogenetic tree of the clade 20A with the cases of B.1.620 lineage in the general hospital of Jeju Island. The tree was constructed using Nextclade. Red circles indicate the genomes from hospital outbreak cases on Jeju Island.
